# Polyarteritis nodosa isolated to the testis and urinary bladder in the setting of cryptorchidism: a case report and literature review

**DOI:** 10.1186/s13256-019-2172-y

**Published:** 2019-07-31

**Authors:** Mohan Stewart, Greg Marcotte, Michael A. Seidman, Natasha Dehghan

**Affiliations:** 1 Department of Medicine, University of British Columbia, Vancouver, Canada; 2 Division of Rheumatology, Department of Medicine, University of British Columbia, Vancouver, Canada; 3 Department of Pathology & Laboratory Medicine, University of British Columbia, Vancouver, Canada

**Keywords:** Polyarteritis nodosa, Testis, Bladder, Single organ vasculitis

## Abstract

**Background:**

Polyarteritis nodosa is a small vessel to medium vessel vasculitis that frequently presents with multi-organ involvement, but can sometimes be limited to single organs such as the testes. Patients often require treatment with glucocorticoids, plus or minus additional immunosuppressive therapy depending on the severity of the disease. We describe a rare case of polyarteritis nodosa involving the right testis and urinary bladder without other systemic features of vasculitis.

**Case presentation:**

A previously healthy 54-year-old First Nations Canadian man presented with intermittent gross hematuria. He underwent surgical excision of his right testis for cryptorchidism and a transurethral resection of a bladder mass. Histology showed an active medium vessel vasculitis in both organs. On extensive clinical, laboratory, and radiographic review, he had no systemic features of vasculitis. On 2-year follow-up, he has not required any systemic therapy and has not developed further symptoms.

**Conclusion:**

Single organ polyarteritis nodosa can sometimes be managed with surgical excision of the involved organ alone. Although our patient had two organs involved, we extrapolated the results of our literature search to guide his care. This case highlights the potential for surgical excision to cure polyarteritis nodosa despite the involvement of two organs in the absence of symptoms and signs of systemic vasculitis.

## Introduction

Polyarteritis nodosa (PAN) is a vasculitis characterized by necrotizing inflammation of medium and small arteries. It can affect many organ systems; most often, it affects the peripheral nerves, skin, gastrointestinal system, muscles, and kidneys. The disease often presents with multi-organ involvement, but cases of isolated organ involvement have been described [1, 2]. A detailed history and physical examination are required to identify features consistent with PAN, such as constitutional symptoms, skin lesions, abdominal pain, and peripheral neuropathy. Once PAN is suspected, a biopsy is usually required to confirm the diagnosis. If a biopsy is not possible, angiography is a reasonable alternative [1]. Treatment is based on the severity of illness and involved organs.

In general, cases of mild PAN can be treated with glucocorticoids alone. More severe cases necessitate the addition of immunosuppressive agents, such as cyclophosphamide. Isolated or single organ PAN can be managed by excision of the organ involved (for example, testis) and serial follow-up to ensure that systemic features do not develop [1, 2].

Our case report explores a rare case of PAN affecting both the testis and urinary bladder.

## Case presentation

**Fig. 1:**
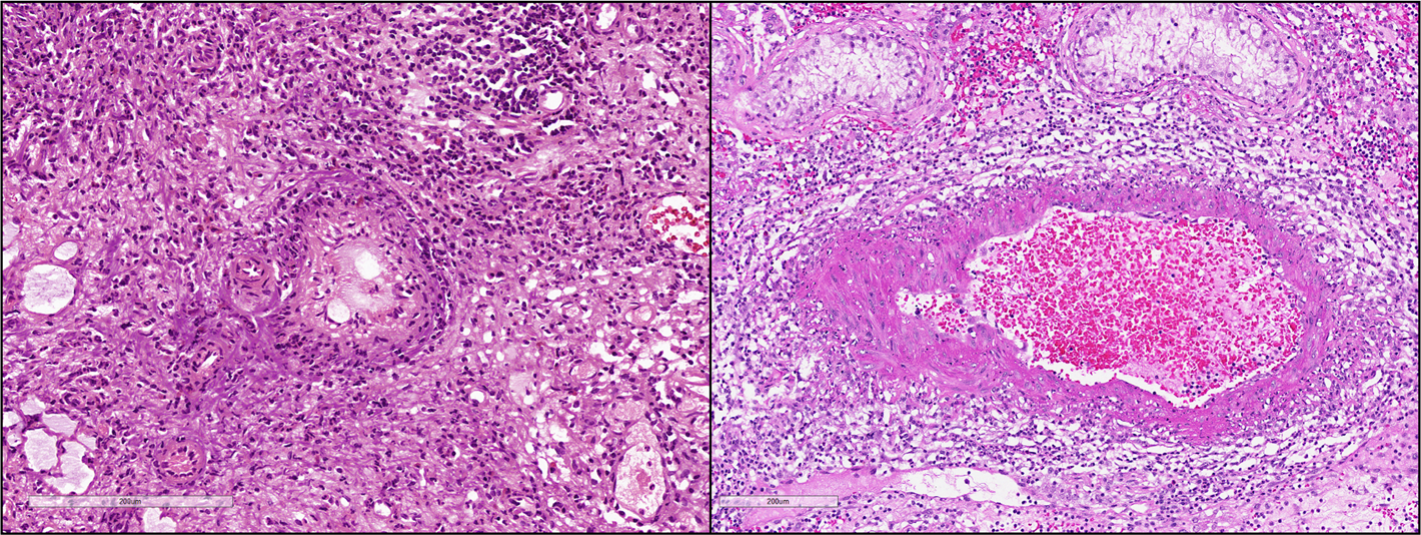
Bladder and testis histology. Hematoxylin and eosin stains. Sections of both bladder (*left*) and testis (*right*) demonstrate active small and medium vessel vasculitis with predominantly mononuclear infiltrate and fibrinoid medial necrosis. Scale bars shown

A previously healthy 54-year-old First Nations Canadian man presented with a several-month history of urinary retention and intermittent gross hematuria. In the course of his initial evaluation, he was found to have an enlarged prostate and was started on tamsulosin with improvement in his urinary symptoms. He noted the absence of his testicle on the right side for at least 30 years, consistent with cryptorchidism. He subsequently underwent cystoscopy, which demonstrated a superficial-appearing mass at the dome of his bladder. A radical right orchiectomy and transurethral resection of the bladder tumor (TURBT) were performed. Surprisingly, a pathologic examination revealed the presence of small and medium vessel vasculitis in both the urinary bladder and undescended testis (Fig. 1). There was no evidence of malignancy. He was referred to rheumatology for further evaluation.

An extensive review revealed no evidence of systemic vasculitis. He had no features of another underlying rheumatologic disorder. His investigations including basic blood work, C-reactive protein (CRP)/erythrocyte sedimentation rate (ESR), and antineutrophil cytoplasmic antibody (ANCA) were normal. Although antinuclear antibody (ANA) was positive (1:320), double-stranded deoxyribonucleic acid (DNA) (dsDNA) and extractable nuclear antigen (ENA) were negative and complements were normal. Rheumatoid factor (RF) was positive at 43 kU/L (reference range < 12 kU/L), but anti-cyclic citrullinated peptides (CCP) antibody was negative and our patient had no features of an inflammatory arthritis. Hepatitis B and C serologies were negative. A diagnosis of PAN was made. His testicular involvement and findings of active small and medium vessel vasculitis on urinary bladder and testis pathology were most consistent with this diagnosis. Given the lack of clinically apparent systemic vasculitis, no immunosuppressive therapy was initiated. Following his radical orchiectomy and TURBT, he had ongoing intermittent hematuria that was investigated with several repeat cystoscopies. An area of erythema was identified on one occasion. A repeat biopsy of the site demonstrated reactive changes and no evidence of vasculitis. A computed tomography (CT) angiogram was done of his abdomen and pelvis to evaluate the possibility of occult vessel involvement elsewhere, and was negative other than wall thickening and fat stranding noted at the anterior bladder. His symptoms have completely resolved following surgery and he has remained asymptomatic over the last 2 years.

## Discussion

To the best of our knowledge, this is the first case reported in the literature of PAN affecting both the bladder and testis in the absence of systemic involvement. Moreover, this is also the first case of PAN reported in the setting of cryptorchidism. We performed a literature search to find reports of patients with isolated testicular or bladder vasculitis and found 45 such cases (Table 1). The average age of presentation was 39 years (median, 36 years). Of the 45 cases, 39 had isolated testicular vasculitis and 6 had isolated bladder vasculitis. Most cases (34/45) had biopsies that were pathologically consistent with PAN.

**Table 1:** Summary of cases of isolated testicular or bladder vasculitis from literature search

Authors	Age/gender	Organ affected	Systemic symptoms	ANCA	ESR/CRP	Pathology	Systemic therapy^a^
Persellin and Menke (1992) [3]	29/M	Testis	None	N/A	N/A	Necrotizing small and medium vessel vasculitis consistent with PAN	None (F/u 17 months)
Halim *et al*. (1994) [4]	71/M	Testis	None	Neg	Normal	Necrotizing granulomatous vasculitis	None (F/u 24 months)
Mukamel *et al.* (1995) [5]	28/M	Testis	None	N/A	Normal	Necrotizing small vessel vasculitis consistent with PAN	None (F/u 27 months)
Mukamel *et al.* (1995) [5]	35/M	Testis	None	N/A	Normal	Necrotizing vasculitis consistent with PAN	None (F/u 36 months)
Kessel *et al.* (2001) [6]	13.5/M	Testis	None	Neg	Elevated	Necrotizing small and medium vessel vasculitis consistent with PAN	None (F/u 24 months)
Hashiguchi *et al.* (2001) [7]	37/M	Testis	None	N/A	Normal	Necrotizing small and medium vessel vasculitis consistent with PAN	None (F/u 9 months)
Eilber *et al.* (2001) [8]	43/M	Testis	Fever, myalgia, and hematuria	N/A	Elevated	Necrotizing vasculitis consistent with PAN (cystoscopy normal)	Not reported (F/u unclear)
Fraenkel-Rubin *et al.* (2002) [9]	26/M	Testis	None	Neg	Normal	Necrotizing small and medium vessel vasculitis consistent with PAN	None (F/u 30 months)
Dotan *et al.* (2003) [10]	32/M	Testis	None	Neg	Normal	Necrotizing medium vessel vasculitis consistent with PAN	None (F/u 60 months)
Tanuma *et al.* (2003) [11]	40/M	Testis	None	N/A	Normal	Necrotizing small and medium vessel vasculitis consistent with PAN	None (F/u 22 months)
Fleischmann and Studer (2007) [12]	21/M	Testis	None	N/A	Normal	Necrotizing small and medium vessel vasculitis consistent with PAN	None (F/u 24 months)
Giannarini *et al.* (2009) [13]	36/M	Testis	None	Neg	Normal	Necrotizing medium vessel vasculitis consistent with PAN	None (F/u 60 months)
Atis *et al.* (2010) [14]	57/M	Testis	None	Neg	Normal	Necrotizing medium vessel vasculitis consistent with PAN	None (F/u unclear)
Saito *et al.* (2013) [15]	78/M	Testis	None	Neg	N/A	Necrotizing vasculitis of epididymis consistent with PAN	None (F/u 12 months)
Lintern *et al.* (2013) [16]	21/M	Testis	None	Neg	Normal	Necrotizing medium vessel vasculitis consistent with PAN	Not reported (F/u unclear)
Garg and Dawson (2015) [17]	36/M	Testis	None	Neg	N/A	Necrotizing small and medium vessel vasculitis consistent with PAN	None (F/u 3 months)
Dixit *et al.* (2017) [18]	84/M	Testis	None	Pos	Elevated	Non-granulomatous medium vessel vasculitis	Not reported (lost to follow-up)
Brimo *et al.* (2011) [19]	35/M	Testis	None	Neg	Normal	Necrotizing small and medium vessel vasculitis consistent with PAN	None (F/u unclear)
Brimo *et al.* (2011) [19]	31/M	Testis	None	Neg	Normal	Necrotizing small and medium vessel vasculitis consistent with PAN	None (F/u unclear)
Brimo *et al.* (2011) [19]	63/M	Testis	None	Neg	Normal	Non-necrotizing granulomatous vasculitis	None (F/u unclear)
Brimo *et al.* (2011) [19]	38/M	Testis	None	Neg	Normal	Necrotizing small and medium vessel vasculitis consistent with PAN	None (F/u unclear)
Brimo *et al.* (2011) [19]	38/M	Testis	N/A	N/A	N/A	Lymphocytic vasculitis	Not reported (F/u unclear)
Brimo *et al.* (2011) [19]	31/M	Testis	None	Neg	Normal	Necrotizing small and medium vessel vasculitis consistent with PAN	None (F/u unclear)
Brimo *et al*. (2011) [19]	53/M	Testis	None	Neg	Normal	Necrotizing small and medium vessel vasculitis consistent with PAN	None (F/u unclear)
Brimo *et al.* (2011) [19]	31/M	Testis	N/A	N/A	N/A	Necrotizing small and medium vessel vasculitis consistent with PAN	Not reported (F/u unclear)
Brimo *et al.* (2011) [19]	23/M	Testis	None	Neg	Normal	Necrotizing small and medium vessel vasculitis consistent with PAN	None (F/u unclear)
Brimo *et al.* (2011) [19]	40/M	Testis	N/A	N/A	N/A	Necrotizing small and medium vessel vasculitis consistent with PAN	Not reported (F/u unclear)
Brimo *et al.* (2011) [19]	34/M	Testis	N/A	N/A	N/A	Necrotizing granulomatous vasculitis	Not reported (F/u unclear)
Brimo *et al.* (2011) [19]	27/M	Testis	None	Neg	Normal	Necrotizing small and medium vessel vasculitis consistent with PAN	Prednisone (F/u unclear)
Brimo *et al*. (2011) [19]	20/M^b^	Testis	Fever, night sweats	Pos	Elevated	Non-necrotizing granulomatous vasculitis	Prednisone, cyclophosphamide (F/u unclear)
Brimo *et al.* (2011) [19]	40/M	Testis	Fatigue	Neg	Elevated	Necrotizing small and medium vessel vasculitis consistent with PAN	Prednisone, MMF (F/u unclear)
Brimo *et al.* (2011) [19]	28/M	Testis	Fever, myalgia	Neg	Elevated	Necrotizing small and medium vessel vasculitis consistent with PAN	Prednisone, cyclophosphamide (F/u unclear)
Brimo *et al.* (2011) [19]	44/M^b^	Testis	Fevers, sweats, weight loss	Neg	Elevated	Necrotizing small and medium vessel vasculitis consistent with PAN	Prednisone, cyclophosphamide (F/u unclear)
Brimo *et al*. (2011) [19]	38/M^b^	Testis	None	Neg	Elevated	Necrotizing small and medium vessel vasculitis consistent with PAN	Prednisone (F/u unclear)
Brimo *et al.* (2011) [19]	34/M	Testis	N/A	Neg	Normal	Necrotizing small and medium vessel vasculitis consistent with PAN	Prednisone, cyclophosphamide (F/u unclear)
Pastor-Navarro *et al.* (2007) [20]	26/M	Testis	None	Neg	N/A	Necrotizing vasculitis consistent with PAN	Steroids (F/u 12 months)
Breuer *et al.* (2015) [21]	19/M^b^	Testis	Erythema nodosum (8 years after initial episode of testicular symptoms)	N/A	Normal	Vasculitis with fibrinoid necrosis consistent with PAN	Prednisone, AZA, rituximab (F/u > 8 years)
Bhatia *et al*. (2018) [22]	52/M	Testis	None	Neg	Elevated	Necrotizing vasculitis consistent with PAN	Prednisone, cyclophosphamide, methotrexate (F/u 8 months)
Islam *et al.* (2018) [23]	41/M	Testis	Polyarthritis, weight loss, rash	Neg	N/A	Necrotizing vasculitis of medium and large vessels consistent with PAN	Steroids, methotrexate (F/u unclear)
Fischer *et al*. (1998) [24]	32/M	Bladder	None	Neg	Elevated	Necrotizing vasculitis of the medium-sized arteries consistent with PAN. Positive for hepatitis B surface antigen immunostaining	TURBT, steroids (F/u 18 months)
Fischer *et al*. (1998) [24]	61/F	Bladder	None	Neg	N/A	Small vessel vasculitis	Steroids, cyclophosphamide (F/u 12 months)
Katz *et al*. (2005) [25]	59/M	Bladder	Fatigue, sweats, weight loss	Neg	Elevated	Necrotizing vasculitis of small and large vessels	Prednisolone (F/u 3 months)
Becker *et al*. (2008) [26]	53/M	Bladder	None	Neg	Elevated	Necrotizing vasculitis of small vessels	TURBT, prednisone, cyclophosphamide (F/u 1 month)
Kassir *et al*. (2013) [27]	31/M	Bladder	Fever	N/A	Elevated	Non-granulomatous, small and medium vessel thrombotic vasculitis	TURBT (F/u 9 months)
Fall *et al*. (2018) [28]	45/M	Bladder	None	Neg	Normal	Fibrinoid degeneration with neutrophil infiltration into vessel walls consistent with vasculitis	TURBT, Prednisone, AZA (F/u 60 months)

*ANCA* antineutrophil cytoplasmic antibody, *AZA* azathioprine, *CRP* C-reactive protein, *ESR* erythrocyte sedimentation rate, *F* female, *F/u* follow-up, *M* male, *MMF* mycophenolate mofetil, *N/A* not applicable, *Neg* negative, *PAN* polyarteritis nodosa, *Pos* positive, *TURBT* transurethral resection of bladder tumor

^a^ All patients with isolated testicular vasculitis underwent orchiectomy

^b^ These patients went on to develop systemic vasculitis or persistent mild cutaneous vasculitis

**Fig. 2:**
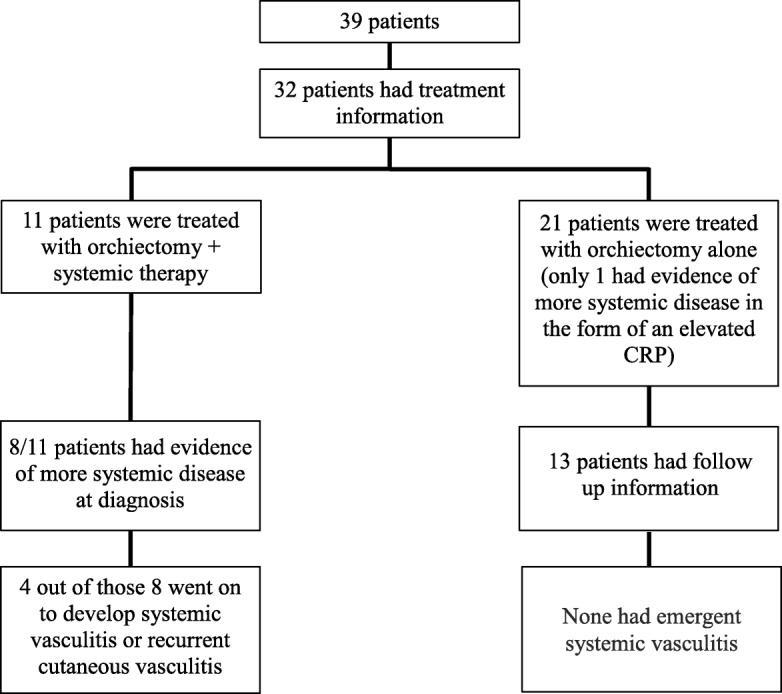
Summary of treatment regimens and outcomes for patients with isolated testicular vasculitis

For the patients with isolated testicular vasculitis in whom treatment information was available, most (21/32) were treated with surgical excision alone with no reported cases of emergent systemic disease over a mean follow-up of 29 months (median, 24 months). Of the 11 patients who were treated with surgical excision as well as glucocorticoids +/− additional immunosuppressive therapy, 8/11 had evidence of more systemic illness at disease onset (for example, elevated CRP/ESR or constitutional symptoms) (Fig. 2). It is unclear what prompted treatment in the other three cases. Three patients were treated with glucocorticoids alone. Eight patients were treated with glucocorticoids in conjunction with at least one other immunosuppressive therapy [19–23]. Of those eight, four were treated with steroids and cyclophosphamide and the other four were treated with steroids in conjunction with cyclophosphamide and methotrexate, mycophenolate mofetil, methotrexate alone, or azathioprine and rituximab.

Of the 11 patients with isolated testicular vasculitis who received systemic therapy, three went on to develop systemic vasculitis [19] and one patient had persistent episodes of mild cutaneous vasculitis [21]. All four patients had evidence of more systemic disease on initial presentation.

Six patients who had isolated bladder vasculitis were included in our literature review. Four of the patients had evidence of more systemic illness at disease onset. The patients who did not have any systemic features underwent either a TURBT as well as therapy with prednisone and azathioprine, or treatment with steroids and cyclophosphamide without surgical excision [24, 28]. Of the other four patients, one underwent TURBT alone [27], two underwent TURBT as well as systemic therapy [24, 26], and one received prednisolone alone [25]. All six patients had no evidence of emergent systemic vasculitis over a mean follow-up of 17.2 months (median, 10.5 months).

It appears from the result of our literature review that patients who present with isolated testicular vasculitis and no evidence to suggest more systemic disease do not go on to develop systemic vasculitis after orchiectomy, regardless of whether or not they receive concomitant immunosuppressive therapy. Therefore, orchiectomy alone in isolated testicular vasculitis seems to be a reasonable therapeutic approach. There were only six patients with isolated bladder vasculitis, and one of them underwent TURBT alone with no emergent systemic illness. We used these findings to guide our management of a patient with PAN affecting both the right testis and urinary bladder.

## Conclusion

This case summarizes the current literature regarding the treatment of isolated testicular or bladder vasculitis. Our findings support withholding immunosuppressive therapy after surgical excision in these patients if there is no evidence of more systemic disease at onset. While single organ PAN has been treated in the past with surgical excision, there is a lack of data regarding treatment of PAN involving two organs, with no other systemic features. We extrapolated the results of our literature review to guide the management of a patient who presented with PAN affecting both the urinary bladder and right testis. He underwent radical right orchiectomy and TURBT, with no systemic therapy. On 2-year follow-up, he has not developed any systemic PAN symptoms. The risk of developing future extratesticular and extravesicular involvement appears to be low.

## Data Availability

Not applicable.
